# Functional regeneration of tendons using scaffolds with physical anisotropy engineered via microarchitectural manipulation

**DOI:** 10.1126/sciadv.aat4537

**Published:** 2018-10-19

**Authors:** Z. Wang, W. J. Lee, B. T. H. Koh, M. Hong, W. Wang, P. N. Lim, J. Feng, L. S. Park, M. Kim, E. S. Thian

**Affiliations:** 1 Department of Mechanical Engineering, National University of Singapore, 9 Engineering Drive 1, Singapore 117 576, Singapore.; 2 College of Materials Science and Engineering, Hunan University, Changsha 410082, P.R. China.; 3 Prestige BioResearch Pte Ltd, 15 Tech Park Crescent, Singapore 638117, Singapore.; 4 College of Veterinary Medicine, Kyungpook National University, Daegu 41566, Republic of Korea.; 5 Department of Orthopaedic Surgery, National University of Singapore, 5 Lower Kent Ridge Road, Singapore 119 074, Singapore.; 6 Department of Electrical and Computer Engineering, National University of Singapore, 2 Engineering Drive 3, Singapore 117576, Singapore.

## Abstract

Structural and hierarchical anisotropy underlies the structure-function relationship of most living tissues. Attempts to exploit the interplay between cells and their immediate environment have rarely featured macroscale, three-dimensional constructs required for clinical applications. Furthermore, compromises to biomechanical robustness during fabrication often limit the scaffold’s relevance in translational medicine. We report a polymeric three-dimensional scaffold with tendon-like mechanical properties and controlled anisotropic microstructures. The scaffold was composed of two distinct portions, which enabled high porosity while retaining tendon-like mechanical properties. When tenocytes were cultured in vitro on the scaffold, phenotypic markers of tenogenesis such as type-I collagen, decorin, and tenascin were significantly expressed over nonanisotropic controls. Moreover, highly aligned intracellular cytoskeletal network and high nuclear alignment efficiencies were observed, suggesting that microstructural anisotropy might play the epigenetic role of mechanotransduction. When implanted in an in vivo micropig model, a neotissue that formed over the scaffold resembled native tendon tissue in composition and structure.

## Teaser

Polymer axial drawing enables the design of optimal microarchitectured scaffolds for tendon regeneration.

## INTRODUCTION

Living cells reside within an intricate milieu of soluble biomolecules, cell-cell interactions, and extracellular matrices (ECM). For most specialized tissues, it is the structural anisotropy and hierarchical organization of the ECM that form the stage for their functions, ranging from the soft (neuron), to moderately stiff (skin, heart, blood vessel, and muscles), to stiff (ligament, tendon, and bone) tissues (*1*, *2*). These complex yet well-defined macro/microarchitectural attributes give rise to the unique functions of these tissues (*3*–*5*). Hence, recapitulating the structure-function relationship of tissues has been a goal for tissue engineering, and multiple studies have emphasized the importance of designing biomaterials with specific microarchitectural structures to direct tissue organization and cell alignment (*1*, *6*–*9*). Among the key principles are designing substrates with various topographies in the range of tens to hundreds of nanometers (*10*, *11*) to engage focal adhesions or in the range of a few tenths of a micrometer (*6*, *9*) to direct individual cells. Altering parameters such as feature arrangement and fiber alignment has shown to have profound effects on cell shape (*12*), growth characteristics (*13*), migration (*14*), and even cell fate (*15*). To date, many studies have featured the use of various topographical geometries and arrangements to achieve specific cell responses (*15*). Despite these advances, clinical translation of these well-established principles persists as a significant challenge. Engineering of highly precise and well-defined microarchitecture onto a biomaterial is often complex and tedious, and the scaffolds with these features are susceptible to surface and material disintegration during physical manipulation. Other attempts to biofunctionalize existing materials with topological features face problems of insufficient porosity in the base material, thus preventing cell migration and blood supply. At present, there exists an unmet need of enabling the translation of microarchitectural principles into actual clinical applications. This need is particularly relevant for solutions involving the repair of force-transmitting tissues such as tendons and ligaments, where the biomechanical demands of these solutions impose the fundamental criterion of high mechanical robustness, which most experimental scaffolds fail to meet. Currently, most medical implants prioritize bulk material properties over microstructural intricacies because, clinically, implant safety and feasibility of application are of paramount concern. For instance, an orthopedic surgeon will favor the use of a rigid steel bone plate to stabilize a complex fracture site over a biomaterial that promises complete bioresorbability and excellent host-tissue regeneration but does not offer the mechanical robustness and strength of a steel plate.

## RESULTS

We reported the development of a novel tendon scaffold, which reconciled the need for a high-strength, highly manipulatable material, while retaining microtopographical features and microarchitectural structures that were appropriate for tendon regeneration. The scaffold comprised two distinct portions: the core and the shell. The core portion consisted of a series of longitudinally aligned electrospun nanofibers, arranged concentrically in a multilayered core. The core was wrapped by a single-layer shell, consisting of a hollow, perforated tube. The perforations were elliptical in shape, elongated longitudinally. By using axial drawing, the scaffold could be fabricated in a simple and scalable manner while having topological (surface ridges and grooves) and microarchitectural (elongated pores and aligned fibers) features appropriate for tendon tissue regeneration. Simultaneously, the polymeric material underwent strain-strengthening, producing a structure with high tensile stiffness.

**Fig. 1:**
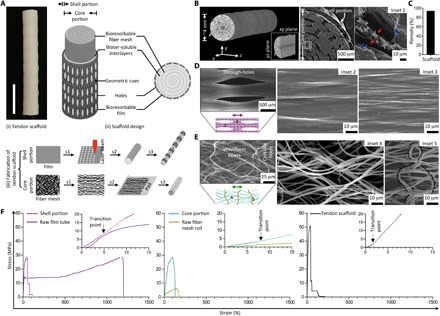
Fabrication of 3D architecture scaffold with controlled anisotropic microstructure and tendon-like mechanical properties. (**A**) Gross appearance of the tendon scaffold designed with core and shell portions building from bioresorbable multilayered PCL fiber meshes and films. Fabrication of the core portion involving steps of uniaxial stretching (s1), layering with a water-soluble poly(ethylene oxide) (PEO) film (s2), and rolling (s3), while the shell portion involving steps of laser microperforation (s1), rolling around a rod to form a hollow tube (s2), and uniaxial stretching (s3). Scale bar, 1 cm. (**B**) Computed tomography (CT) three-dimensionally (3D) reconstructed and scanning electron microscopy (SEM) cross-sectional images of the tendon scaffold that shows a well-assembled multilaminar structure, with a single film layer constituting the shell portion and multiple fiber mesh layers (separated by PEO interlayers) constituting the core portion. Red and blue arrows (inset 1) representing the fiber and PEO interlayer, respectively. (**C**) Calculated porosity of the tendon scaffold after dissolving PEO interlayers. (**D**) SEM images of the shell portion that shows elongated through-holes and orientated ridges/grooves in bulk regions (insets 2 and 3) toward stretching direction (double-headed arrow). (**E**) SEM images of the core portion that shows orientated waveform fibers (inset 4) toward stretching direction (double-headed arrow) and relatively thicker fibers (inset 5; inactive dashed line) across stretching direction. (**F**) Representative stress-strain curves of the shell portion, core portion, and tendon scaffold showing reinforced material fabrication. Raw film tube and fiber mesh roll representing control groups of the scaffold shell and core portions, respectively.

To form the shell portion, a bioresorbable poly(ε-caprolactone) (PCL) film first underwent laser perforation to create round through-holes. Opposite edges of the film were rolled to meet each other, forming a hollow tube through heat sealing, which underwent controlled thermal drawing longitudinally to create pores and surface structures elongated along the longitudinal axis (Fig. 1A). The core portion was prepared via cold axial drawing of an electrospun PCL mesh, followed by concentric rolling with a water-soluble PEO film. Figure 1B showed micro-CT images capturing the 3D architecture of an as-fabricated scaffold with ~4 mm in diameter, which exhibited well-assembled laminae throughout. SEM images of the scaffold cross section demonstrated that the laminae in the shell and core portions were packed closely together. After complete dissolution of the PEO interlayers, the scaffold had a high porosity of ~94%, thus satisfying the requirement for adequate neotissue ingrowth (>90%; Fig. 1C) (*16*, *17*). Pore size, as shown in Fig. 1 (D and E), was also much larger than the size of cells (~1 to 10 μm), thus enabling the migration of cells from the shell into core portion of the scaffold.

SEM images of the shell portion (Fig. 1D) showed series of hole and nonhole regions, regularly spaced, to enable transverse mass transportation throughout the scaffold while retaining mechanical integrity. The nonhole regions featured microridges/grooves aligned along the stretching direction. To investigate the dynamics of PCL deformation, we varied draw ratio (initial length/final length) between 1 and 5 (figs. S1, A to D). At a draw ratio of ~2, the nonhole regions experienced preferential deformation along the stretching direction, with the formation of necks accounting for the increased short axis of holes. Meanwhile, the necks extended during axial drawing and resulted in enlargement to the long axis of holes. Further drawing (draw ratios of 3 to 5) resulted in neck expansion to the left regions, which reduced the tubular diameter of the shell associated with significant reduction in the hole’s short axis and continuous increase in the hole’s long axis. The formation of microridges/grooves correlated closely to polymer shoulder propagation. Stretching at higher draw ratios tended to result in increased ridge length, with the inter-ridge distance declining initially and then rising subsequently. Thus, the differences between microridges/grooves at interhole regions along the stretching direction to those across were due to the effects of local deformation (Fig. 1D). Sufficient drawing of the scaffold (for example, at a draw ratio of 5) allowed for full coverage of microridges/grooves and consistent hole geometries.

SEM images of the core portion revealed a porous architecture of mostly wave-like fibers aligned longitudinally, with transverse interlinking fibers connecting the longitudinal fibers at regular intervals (Fig. 1E). Alignment of the fibers was created through cold axial drawing, which was distinct from aligned fibers generated by electrospinning (fig. S2, A to C) (*18*). At small draw ratios (1 to 2), fiber displacement within the mesh occurred so as to tighten the majority of fibers, while at the same time relaxing the remaining fibers. The tightened fibers reorientated gradually along the stretching direction and formed necks locally to propagate until the fibers were fully drawn, while the relaxed fibers tended to organize across the stretching direction without necking. Further drawing (a draw ratio of ~5) resulted in formation of wave-like fibers, which enabled the fibers to space out while retaining fiber alignment. As a result, the presence of interfibrillar spacing could allow the cells to reside among the fibers. This represented an advantage over other aligned fibers formed by conventional electrospinning, in which mechanical pulling of the fibers as it was electrospun created highly dense fibers with significant fiber staking and limited pore size and porosity, hence eliminating the appropriate interfibrillar spacing required for proper cell attachment and growth (*18*, *19*).

Morphological examination suggested that the scaffold’s design resembled the hierarchical and anisotropic structure in tendon tissue. To investigate whether it exhibited similar modulus and compliant biomechanical behavior to native tendon (*5*), we conducted mechanical characterization. Stress-strain curves of the shell following tensile deformation showed constant stress increase until failure (Fig. 1F). This was related to the axial strain-strengthening effect of the polymer. Compared to the nonstretched version, the shell exhibited higher yield stress and comparable Young’s modulus and yield strain (Table 1). This was attributed to the effects of strain strengthening, compensating for the weakening effect of hole enlargement (table S1) (*20*). Stress-strain curves of the core showed similar strengthening phenomena due to polymer axial drawing, as evidenced by significant improvement in the Young’s modulus, yield stress, and yield strain (Fig. 1F and Table 1).

**Table 1:** Mechanical properties of tendon scaffold. (*n* = 4).

**Samples**	**Young’s modulus (MPa)**	**Yield stress (MPa)**	**Yield strain (%)**	**Ultimate stress (MPa)**	**Break strain (%)**
Raw film tube	225.4 ± 9.5	12.4 ± 1.6	11.5 ± 2.4	37.4 ± 6.6	1457.6 ± 206.1
Shell portion	197.7 ± 37.2	18.6 ± 2.0	11.1 ± 1.3	27.3 ± 3.6	58.9 ± 8.2
Raw fiber mesh roll	18.5 ± 1.2	1.4 ± 0.3	8.2 ± 1.9	6.0 ± 0.7	152.6 ± 4.5
Core portion*	51.0 ± 8.9	22.7 ± 3.0	42.5 ± 5.3	32.9 ± 2.1	148.9 ± 23.5
Tendon scaffold*	374.9 ± 66.8	38.4 ± 6.6	12.2 ± 0.8	56.2 ± 6.0	47.5 ± 3.6
Human patellar graft (*4*, *22*)	~191–660	/	/	~26–95	~13–31

*Samples dissolving off PEO interlayers.

Stress-strain curve of the tendon scaffold exhibited mechanical performance similar to that of the human tendon (*5*). This was characterized by a nonlinear “toe region,” followed by a linear stress increase within a very short strain range before failure. The toe region functioned to buffer sudden tensile strain, which was typically in the range of 1 to 3% for native tendons (*21*). Quantitatively, the tendon scaffold had increased mechanical strength as compared to its individual components. The measured Young’s modulus approached that of the human patellar tendon graft (~191 to 660 MPa) (*4*, *22*) and was superior when compared to most scaffolds featuring collagen, poly(lactic acid)- and poly(lactic-co-glycolic acid)-based knitted (~7 to 67 MPa) (*23*, *24*), braided (~55 MPa) (*25*), and core-shell (~1 to 8 MPa) (*16*, *26*) types. Furthermore, the measured ultimate stress and breaking strain of the scaffold approached the reported values (~26 to 95 MPa and ~13 to 31%, respectively) of human patellar tendon graft (*4*, *24*). This might suggest that the scaffold could avoid inelastic deformation and catastrophic failure under physiological loads after implantation. Such a claim would require more in-depth in vitro and in vivo studies to verify.

**Fig. 2:**
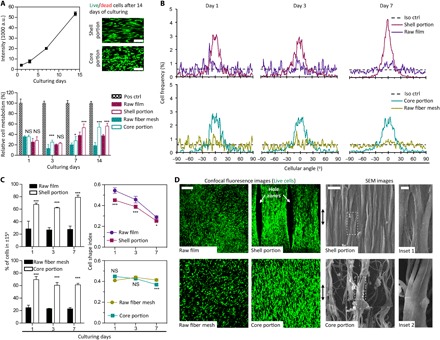
Interfacial cytocompatibility and morphological induction of human tenocytes with anisotropic architecture of the tendon scaffold. (**A**) Metabolic analysis and confocal images of human tenocytes that show proliferative cell growth without inducing interfacial cytotoxicity in the tendon scaffolds (green, live cells; red, dead cells). Pos ctrl, tissue culture plate as positive control; raw film tube, control of the shell portion; raw fiber mesh roll, control of the core portion; a.u., arbitrary units. Scale bars, 100 μm. [*n* = 6; *P < 0.05, ***P < 0.001, and not significant (NS; *P* > 0.05) based on Student’s paired *t* test with two-tailed distribution]. Error bars represent SD. (**B**) Representative cell angular frequency curves of human tenocytes that, during proliferation in the tendon scaffold, tend to organize toward a preferential direction. Iso ctrl, isotropic control having a value of 0.56%. (**C**) Cell alignment and elongation analysis of human tenocytes that is described as percent number of cells with angles in ±15° and cell shape index (CSI), respectively. (*n* = 3; *P < 0.05, ***P < 0.001 and NS *P* > 0.05 based on Student’s paired *t* test with two-tailed distribution). Error bars represent SD. (**D**) Representative confocal fluorescence and SEM images of human tenocytes after 14 days of culturing forming into cell bundles to align along axial architecture of the tendon scaffold (green, live cells). Double-headed arrow, scaffold longitudinal axis. Scale bars, 200 μm (confocal image), 10 μm (SEM image), and 5 μm (inset).

To understand whether geometric features created from polymer axial drawing could stimulate tenogenesis, we seeded adult human tenocytes in both portions individually and in the assembled construct for in vitro biological evaluation (see Materials and Methods). It was found that the scaffold offered the cells a conducive microenvironment for proliferation, demonstrating sustained metabolic increase over ~2 weeks of culturing (Fig. 2A). Compared to the isotropic control group of raw PCL film, tenocytes cultured on scaffold’s shell portion exhibited higher metabolic activity as early as day 3 of culture. For the scaffold’s core portion, tenocytes showed higher metabolic activity on day 7 onward, as compared to the raw fiber mesh group. By day 14, tenocytes cultured on the shell (2.9× of the raw film group, *P* < 0.001) and core (1.5× of the raw fiber mesh group, *P* < 0.001) portions exhibited greater metabolic activities as compared to their isotropic control groups, respectively. The shell and core portions of the scaffold had physical anisotropy that mimicked a tendon’s ECM. This key difference, compared with PCL controls without physical anisotropy, might have accounted, to some extent, for the improved total cell metabolism after 1 week of culturing. Confocal fluorescence images obtained after 2 weeks of culturing revealed no presence of dead cells, with presence of live cell populations migrating into the core portion.

To elucidate the role of the tendon scaffold’s geometric features on influencing cell morphogenesis, we conducted individual cell alignment measurements among the study groups. In both the tendon scaffold’s shell and core portions, frequency distribution of cell angles showed a preferential orientation for tenocytes to organize in the longitudinal direction, while we observed a ubiquitous distribution of cells in the control groups (Fig. 2B). This aligned cell organization was established as early as day 1 after culturing and retained after 7 days of in vitro culture. The geometric features of the scaffold resulted in ~60% of cells aligning ±15° to the desired direction, with cells adopting highly elongated, spindle-like morphologies after prolonged culturing periods (Fig. 2C). At day 7, cells cultured on the shell portion exhibited a 1.9× (*P* < 0.001) greater alignment efficiency (percentage of cells in ±15°) over the group of raw PCL film and a 1.7× (*P* < 0.001) higher alignment efficiency for the cells cultured on the core portion as compared to the group of raw fiber mesh. This was further supported by lower CSI measurements over the controls after 1 day for cells cultured on the shell portion and 7 days for cells in the core portion. A lower CSI corresponds to a more elongated cell shape, whereas a high CSI represents a more rounded cell shape. Confocal fluorescence images of tenocytes obtained after 2 weeks of culturing demonstrated organized distribution of confluent cells assembling into bundles (essential units for collagen matrix production) in both the scaffold’s shell and core portions, with the orientation of alignment and shape elongation directed along the geometric features of the scaffold (Fig. 2D). In contrast, cells in the control group exhibited random self-organization, atypical of tissue organization in the native tendon. Thus, these results showed that both shell and core portions of the scaffold tended to have aligned and more elongated cell shapes compared to their respective controls, indicating that the anisotropic microarchitecture of the scaffold is responsible for the differences.

**Fig. 3:**
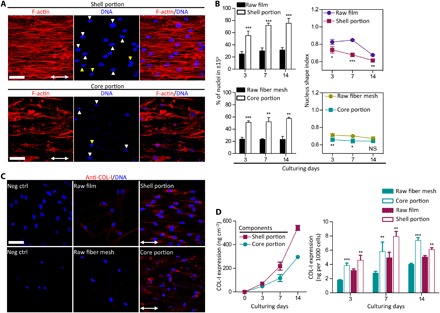
Instructive nucleus deformation and tenogenic matrix expression in the tendon scaffold. (**A**) Representative confocal fluorescence images of human tenocytes that show cytoskeletal organization and nucleus morphology in the tendon scaffolds after 14 days of culturing. Red, F-actin; blue, DNA. White arrowhead, nucleus distortion along central fibers; yellow arrowhead, nucleus distortion away from central fibers; double-headed arrow, scaffold longitudinal axis. Scale bars, 50 μm. (**B**) Nucleus alignment and elongation analysis of human tenocytes that is described as percent number of nuclei with angles in ±15° and nucleus shape index (NSI), respectively. Cells cultured in the shell and core portions of the tendon scaffold obtain higher efficiencies of nucleus alignment and elongation than controls. (*n* = 3; *P < 0.05, **P < 0.01, ***P < 0.001, and NS *P* > 0.05 based on Student’s paired *t* test with two-tailed distribution). Error bars represent SD. (**C**) Representative confocal fluorescence images of human tenocytes that express elevated collagen type I (COL-I) (the major tendon matrix protein) in the tendon scaffold shell (versus control of original film tube) and core (versus control of original fiber mesh roll) portions. Red, COL-I; and blue, DNA. Double-headed arrow, scaffold longitudinal axis. Neg ctrl, negative control. Scale bar, 50 μm. (**D**) Quantitative measurement of COL-I that shows lasting secretion for human tenocytes in the tendon scaffold, at up-regulated levels than controls. Original film tube, control of the shell portion; original fiber mesh roll, control of the core portion. (*n* = 4; **P < 0.01 and ***P < 0.001 based on Student’s paired *t* test with two-tailed distribution). Error bars represent SD.

Geometric features were proposed to deliver epigenetic cues via mechanotransduction, whereby certain morphological configurations of the cell resulted in a combination of both cytoskeletal and nucleus alterations, which then regulated the activity of specific genes in the cell (*19*, *27*, *28*). Confocal fluorescence images of tenocytes stained with actin and DNA revealed organized arrangement of cytoskeletal actin filaments in cells found on the scaffold’s shell and core portions (Fig. 3A). This was closely associated with cell nucleus alignment. In the control groups, the unorganized actin filaments were associated with random nucleus alignment (fig. S3). The relationship between the actin filaments and nuclei might be modeled by subfamilies of actin filaments known to influence nuclear distortion (*9*). The deformation of nuclei as shown in Fig. 3B established a close correlation among actin filaments, cell morphology, and nuclear shape. Determining the subclasses to which these actin filaments belong would require further in-depth study, but it was evident that alignment of these actin fibers was closely associated with high nuclear alignment. Nucleus alignment efficiency (percentage of nuclei in ±15°) of cells cultured on the shell and core portions of the scaffold showed significant enhancements as compared to their respective controls. Furthermore, the nucleus alignment efficiency of cells was 55.1 and 57.6% for the shell and core portions at day 3 of culture, respectively. This efficiency was further maintained throughout the 14 days in culture, with 1.4× greater for the shell (versus the group of raw PCL film, *P* < 0.001) and 1.5× greater for the core (versus the group of raw fiber mesh, *P* < 0.001) portions. Nucleus deformation has been shown to regulate genes through alterations to the transcription factor affinity of DNA (for example, chromatin condensation), nuclear matrix organization, and nuclear pore complex for transportation (*9*, *27*). Coupled with the low NSI, this seemed to suggest that cytoskeletal and nuclear alignment were the factors for up-regulation of COL-I (Fig. 3C).

COL-I is the most major protein forming tendon matrix, and particularly, the spatially and hierarchically aligned organization of COL-I fibril is the prominent feature for mature tendon functions (*26*). As observed in the in vitro study, the expression of COL-I was presented in an anisotropic manner, whereby COL-I epitopes in both the shell and core portions were aligned parallel toward the scaffold’s longitudinal direction. Coupled with the observed aligned cell bundles at the core and shell portions, this organization, as opposed to a random, homogeneous distribution of COL-I, could be an indication of tendon fibrillogenesis (*29*). These observations were absent in the nonanisotropic films or meshes, which in turn did not result in a high expression or alignment of COL-I. Decorin (DCN) is the most abundant small leucine-rich proteoglycan in tendon and plays a critical role in ensuring correct alignment and stabilization of collagen fibrils that is associated with tenogenic phenotype. The inhibition to DCN expression in tenocytes resulted in phenotypic fidelity, which led to a random and disorganized structure of COL-I fibrils, weaker biomechanics, and poor tendon healing (*30*). As shown in fig. S4A, tenocytes seeded on both the core and shell portions of the scaffold exhibited a higher DCN expression over the nonanisotropic controls groups, with no indication of degeneration in the tenogenic potency. Tenascin-C (TNC) is a characteristic tendon ECM constituent that is featured as a tenogenic phenotype marker in previous studies (*31*, *32*). As TNC plays an important role during tenogenesis (for example, to mediate cell-to-substrate adhesion, cell migration, tissue alignment and organization, and the remodeling of tendon tissue), its presence would be associated with proper tenogenic potency. In parallel with COL-I and DCN, a higher expression of TNC was also observed in tenocytes when cultured on the scaffold, and this suggested that a more appropriate tenogenic response (no de-differentiation) was proceeding as compared to the respective controls (fig. S4B).

These results suggested that the physical anisotropy of geometric features had the potential of activating tenogenic pathways. As evidenced, tenocytes cultured on the tendon scaffold for 14 days had been found with prolonged production of COL-I (Fig. 3D). This COL-I production for the cultured cells in the shell portion was 1.2 to 1.5× of the raw film group (*P* < 0.01) and 1.8 to 2.1× of the raw fiber mesh group (*P* < 0.01) for the cells in the core portion. This COL-I production correlated closely to NSI over the investigated period, and an intermediate NSI (0.61 < NSI < 0.73) for tenocytes in scaffold shell generated maximum synthesis of COL-I (Fig. 3, B and D), potentially suggesting the existence of an optimal nucleus distortion that affected tenogenic gene regulation (*28*). An optimal NSI for tenocytes during the investigated period was not evident in scaffold core, indicating that fibrous microarchitectures might have played a greater role in COL-I gene regulation.

**Fig. 4:**
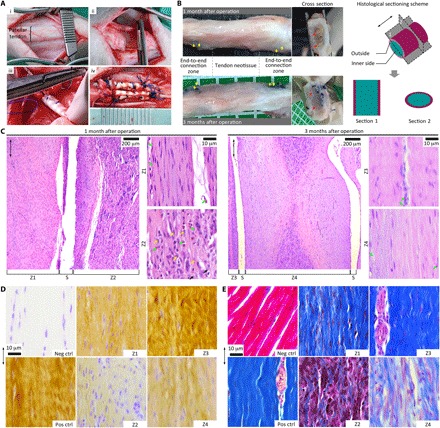
Reconstruction of tendon neotissue. (**A**) Establishment of a tendon injury model. (i) Patellar tendon in the hind limb of micropig, (ii) defect gap (2 cm), (iii) end-to-end grafting, and (iv) tendon scaffold to bridge cutting ends. (**B**) Gross appearance of neotendon tissue construct harvested 1 and 3 months after operation. Yellow arrow, surgical line; red arrow, implanted scaffold; double-headed arrow, longitudinal axis of the neotendon construct. Outside: Tissue growing out of the scaffold; inner side: tissue growing into the scaffold. (**C**) Histological images of neotendon construct after H&E staining showing gradual neotissue formation and vanishing of cell populations. Pink, tissue matrix; purple, cytoplasm; blue, nuclei. Double-headed arrow, neotendon longitudinal axis; yellow arrow, prominent nucleoli; green arrow, blood cell and vessel; s, scaffold’s edge; Z1 and Z2, outside and inner zones of scaffold 1 month after implantation; and Z3 and Z4, outside and inner zones of scaffold 3 months after implantation. (**D**) Immunohistological images of neotendon construct showing progressive deposition of collagen matrix with prolonged implantation from 1 to 3 months. Yellow, COL-I; blue, nuclei. Neg ctrl, negative control labeled with isotype immunoglobulin G1 (IgG1); Pos ctrl, native tendon tissue as positive control. Double-headed arrow, tendon longitudinal axis. (**E**) Histological images of neotendon construct after Masson’s trichrome staining demonstrating fibrous structure formation. Blue, collagen fibers; red/pink, cytoplasm; dark purple, nuclei. Neg ctrl, heart tissue as negative control; Pos ctrl, native tendon tissue as positive control. Double-headed arrow, neotendon longitudinal axis.

While the provision of appropriate microarchitectural features is a requirement for proper cellular and tissue organization, the macrostructural design of the scaffold construct is crucial toward ensuring overall structural robustness, stability, and biomechanical function. To this effect, the relevance of such a scaffold in clinical applications was demonstrated in an in vivo micropig injury model, whereby a full defect (2 cm in gap) was created in patellar tendons of the two hind limbs (Fig. 4A). Videos documented the condition of the micropigs at 1 month after operation (cast was being removed), whereby micropigs could perform activities such as standing, walking, and running, without the use of any external fixation devices (movie S1). Implants harvested at this time point demonstrated partial neotissue formation at the defect position, with typical tendon-like glistening white appearance (Fig. 4B) (*33*). At 3 months, the tissue sample extracted had a clearer appearance, with an indication of some neotissue progressing toward maturation.

For histological analysis, we sectioned the samples along the longitudinal and transverse planes. Hematoxylin and eosin (H&E) staining of implants 1 month after operation showed partial formation of organized neotissue along the scaffold longitudinal axis (Fig. 4C), the high cell nuclei density in the core portion could possibly be associated with the fibroplasia phase, while the dense tissue structure of the shell portion could correspond to the remodeling phase of tissue healing. The interconnected porosity of the tendon scaffold might have allowed for abundant cellular and blood infiltration, while the structural components of the scaffold might allow for cellular attachment and tissue deposition. In contrast to the amorphous organization of cells and tissues in scar tissue formation (*26*), the cells and partial neotissue developed in an organized and aligned manner, as directed by the scaffold architecture. Immunohistochemistry images of this neotissue construct exhibited positive and aligned COL-I deposition, while the differing intensities of staining at the shell portion and core portion corresponded to the different healing phases (Fig. 4D).

At 3 months after operation, healing progressed across the scaffold (Fig. 4C). Longitudinally, the neotissue near the shell portion showed tendon-like waveform structure, indicating partial neotissue maturation, while the newly formed tissue at the core portion underwent remodeling to achieve a well-aligned cell and matrix morphology propagating gradually from the scaffold edges. Transversely, H&E images of the neotissue cross section further demonstrated possible tissue compartmentalization similar to the native tendon, and evidence of partial mature fascicle formation near the shell portion (a dense hypocellular structure surrounding by a looser hypercellular tissue), accompanied by infancy fascicle at the core portion (fig. S5A). Moreover, prolonged implantation resulted in neotissue consolidation and significant reduction in cellularity, which associated with greater deposition of COL-I in both the shell and core portions of the scaffold at 3 months after operation (Fig. 4D and fig. S5B). Coupled with this, histological Masson’s trichrome staining showed fibrous structure formation of the deposited COL-I, with differing fibrosis intensities in neotissue at differing maturation degrees (Fig. 4E and fig. S5C). During the formation of the neotissue construct, the existence of multiple healing phases at progressive phases of maturation from the shell to the core portion suggested the necessity for tendon scaffold design to have high porosity so as to allow for tissue remodeling and maturation processes to progress while facilitating the transport of nutrients to and within the core portion of the scaffold.

## DISCUSSION

Conventional surgical treatments including tenorrhaphy and augmentation are still prone to reruptures and graft failure. In addition, despite the existence of commercially available products, there is still a missing gap in innovation to create a product, which has superior biomechanical strength and, at the same time, allowing tendon regeneration. At present, organically derived reinforcement patches (for example, Stryker’s TissueMend and Wright’s GraftJacket) are used as a wrap-around to the primary suture and are not designed to sustain any major mechanical loads. The resultant tissue that grows over these patches may not resemble the structure nor the composition of the native tendon. Meanwhile, currently commercially available products for the treatment of tendon ruptures [for example, Restore Orthobiologic Soft Tissue Implant (DePuy Synthes, Johnson & Johnson) and CollaFix (MiMedx)] are derived from bovine sources, and this represents a potential risk of host tissue immunogenic inflammation.

The featured tendon scaffold, being fully synthetic, did not have any biological material, and the base biomaterial PCL has been proven to have excellent biocompatibility with the host tissue in several U.S. Food and Drug Administration–approved applications. The results from this study thus far demonstrated that the techniques used in the fabrication of a macrosized PCL scaffold, composed of two distinct but complementary portions, could help in the recreation of tissues where high strength and anisotropy were required (for instance, the tendon). Instead of attempting to replicate tissue structure, the scaffold was developed to apply the principle of incorporating physical characteristics in the material to establish key interactions with cells, which facilitated and guided the regeneration process (*1*, *34*). In line with this hypothesis, it was found that geometric features generated through cold axial drawing might be able to deliver microtopological and microarchitectural cues to influence tenogenesis in vitro and in vivo. Furthermore, the engineering of these features in a macrosized construct need not result in adverse compromises to the biomaterial’s structural and mechanical properties.

Looking forward to the next generation of techniques involved in the fabrication of anisotropic scaffolds, a few notable sources could be observed. These were the pressurized gyration that involved rotating a perforated pot at high speed, which contained a polymer solution in a solvent, as described by Hong *et al*. (*35*), and a nonsolvent, as reported by Xu *et al*. (*36*). These techniques represented a marked advance over the most common methods in producing polymeric fibrous materials and had the advantages of mass fabrication of highly well-aligned fibers, unlimited selection of materials, and low cost. Comparatively, the present paper described a method of combining common electrospinning and axial drawing that was as simple and cost-effective as the pressurized gyration technique in producing aligned fibers. Another difference was that this technique further had unique properties of generating the wave-like aligned single fibers, which tended to mimic the geometry of fibrils in the native tendon, as well as elevated interfibrillar spacing that would be important for cells to reside among the fibers, and facilitate vessel and tissue formation. In addition, the process of the axial drawing also incorporated strain-induced enhancement that could enable the wave-like aligned fibers to have improved mechanical performance, which would be crucial for the repair of force-transmitting tissue such as tendons.

The scaffold development in this study was distinctly different from the fibrous scaffold reported by Li *et al*. (*37*), which created structure anisotropy through the deposition of electrospun fibers on a rotating device for musculoskeletal tissue engineering. The primary aim of producing aligned fibers was to create cell alignment, and it was not meant to be suitable to withstand load bearing for the repair of force-transmitting tissues such as tendons. Recognizing this fact, the scaffold core portion exhibited much higher yield stress, yield strain, and ultimate tensile stress as compared to the nonstretched fiber, by using single-axial drawing. In addition to the strain-induced enhancement, the tendon scaffold was engineered with the fibrous core portion wrapped by a single-layer shell to provide mechanical support and bear most of the loading. These made the aligned fibers more relevant for tendon tissue–engineering applications. In comparison with other studies reported on electrospun scaffolds for musculoskeletal tissue engineering, the tendon scaffold featured improved mechanical properties as compared to a coelectrospun tendon-muscle scaffold reported by Ladd *et al*. (*38*).

We have shown that the tendon scaffold might provide a microenvironment conducive for cell growth, fostering appropriate cell alignment and tissue organization. We have also shown that the tendon scaffold had mechanical properties superior to those conventional scaffolds close to that of the native patellar tendon. Thus, these properties might be more appropriate for use in the repair of human tendons, allowing the injured tendon to be more robust during the recovery period (that is, better injury stabilization and improved force transmission). Moreover, it would give rise to better long-term outcomes due to complete bioresorbability of the scaffold while ensuring that the tissue, which took over the scaffold, was identical to the native tendon in structure, composition, and function. Certainly, these assessments were preliminary, and therefore, more in-depth studies of the tendon scaffold’s mechanical properties (for example, with cyclic testing under physiological loads) would be planned as a follow-on study to obtain the fatigue, creep, and hysteresis characteristics of the tendon scaffold, with direct comparison with cadaver tendon samples. In vivo studies with statistically significant sample sizes and various control groups shall also be used. As this is the first study conducted, assessments regarding the scaffold’s mechanical robustness and biocompatibility suggested that it was safely implanted in an animal model. This was in fact confirmed, 1 month after implantation, when animal subjects were able to perform normal gait and even run without complications. Nevertheless, a full in vivo study, in which a larger animal (for example, a horse) with more statistically significant numbers, would be needed for future work to gain more quantitative analyses of biomechanics, biocompatibility, and tendon tissue regeneration.

This study contributed to scaffold development and featured microscale 3D structural and hierarchical anisotropy to direct desired biological response, while being highly porous yet mechanically robust for tendon repair applications. Overall, our results demonstrated the potential feasibility of using specific engineering techniques for the creation of macrosized scaffolds relevant for tendon repair. These scaffolds had specific geometric features, which delivered physical cues in a spatial manner that might lead to appropriate tissue regeneration. These techniques might be further applied to the other scaffolds requiring specific geometric features for the construction of tissue-specific functional architecture, thus advancing the translation of regenerative therapies for soft tissue repair.

## MATERIALS AND METHODS

### Scaffold preparation

The shell and core portions of the tendon scaffolds were fabricated using separated protocols. The fabrication of the shell portion involved laser perforation, rolling, and mechanical stretching. Briefly, PCL [molecular weight (*M*_w_) = 80,000; Sigma-Aldrich] pellets underwent two-roll milling and was heat pressed (80°C, 30 MPa, 24 hours) into a raw film (~120 μm in thickness). This film was perforated, as reported previously using a CO_2_ laser (wavelength, 10.6 μm and repetition, 10 kHz; Access Laser, USA) (*39*, *40*). Laser beam tracks were controlled using CyberLase software (IDI Laser Services Pte Ltd, Singapore), which produced holes (~120 μm in diameter) at an interhole distance of 750 μm. The laser-perforated film was rolled around a rod, forming a hollow tube (6 mm in diameter). Thereafter, the ends were heat sealed, and the tube further underwent stretching (54°C; draw ratio, 5) along the longitudinal axis using an in-house–developed machine.

The fabrication of the core portion involved mechanical stretching, layering, and rolling. Briefly, a raw mesh of PCL fibers (~2.7 μm in diameter) was obtained from electrospinning, as reported previously (*41*). Axial stretching of the fibers was then performed (room temperature; draw ratio, 5) using an in-house–developed machine. The stretched fiber mesh was laid onto a PEO film (*M*_w_ = 200,000 and ~100 μm in thickness; Sigma-Aldrich) and rolled across the direction of axial stretching, where PEO film formed helical interlayers occupying most of the scaffold inner space. The tendon scaffold was assembled by inserting the core portion into the shell portion and secured tightly by relaxing the core portion.

### Material characterizations

The as-fabricated tendon scaffold was examined using a field emission SEM (S-4300, Hitachi, Japan) and micro-CT (1076, Bruker SkyScan, USA). Briefly, scaffolds were sputter gold coated (10 mA, 30 s) and imaged using SEM at an accelerating voltage of 15 kV. Images were analyzed using the built-in function of ImageJ software [National Institutes of Health (NIH), USA] for structural parameters of the shell (ridge length, inter-ridge distance, and through-hole size) and core (fiber angle and diameter) portions prepared at various draw ratios (1, 2, 3, 4, and 5). Three distinct replicates were tested for the shell (a total of 8 to 20 holes and 40 to 60 ridges analyzed) and core (a total of 80 fibers analyzed) portions at each draw ratio. The as-fabricated scaffold was scanned by micro-CT at a pixel size of 9 μm and reconstructed using VGStudioMax software (Volume Graphics GmbH, Germany). For detailed examination, sections across the scaffold’s long axis were prepared in liquid nitrogen and imaged using SEM.

Mechanical properties of the as-fabricated tendon scaffold were evaluated using a tensile testing machine (3345, Instron, USA). Briefly, PEO was removed from the tendon scaffold by dissolving in a phosphate-buffered saline (PBS) buffer. The dimension (width, length, and thickness) of the dried sample was measured using a digimatic micrometer (APB-1D, Mitutoyo, Japan). Tensile testing was performed at a load cell of 100 N and a pulling rate of 10 mm min^−1^, as reported previously (*12*, *42*). PCL tube from raw film (as the control of shell portion) and raw fiber mesh (as the control of core portion) were also studied for comparison. A low yield point was used for the control of shell portion to determine the yield stress and strain at linear-elastic regions, while an offset strain of 0.5% was used for other types of testing samples to determine their yield points. Four replicates were tested for each sample.

### Cell source and culture

Human tenocytes (CSC-C1584) were obtained from Creative Bioarray (USA) and cultured in an HTGM-500 growth medium (Creative Bioarray) according to the recommended protocols. The culture media were changed every 3 days, and cells were passaged at nearly 90% confluence using 0.25% trypsin (Gibco). Tendon scaffolds (with alkaline treatment) were sterilized using 70% ethanol (12 hours) and then washed thrice with PBS for in vitro cell culture investigation. Cells at passage 6 were used in this study.

### Fluorescence and immunofluorescence

The cell metabolism was examined using an alamarBlue assay (Thermo Fisher Scientific, USA), as reported previously (*43*). Briefly, human tenocytes (10,000 cells in 200 μl) were seeded onto the as-fabricated tendon scaffold (sample size, 1 cm in length and 4 mm in diameter) in low-adhesion 24-well plates (BD Biosciences, USA). Cells were incubated for 6 hours to allow initial attachment and cultured with the addition of another 800 μl of growth medium for proliferation. For the analysis of cell metabolic levels, the culture medium was replaced by 10% alamarBlue medium (1 ml per well) and incubated for 4 hours. The fluorescence of supernatant medium was read at 560/590 nm (excitation/emission wavelength) using a Microplate Reader (Synergy H1, BioTek, Singapore) at 1, 3, 7, and 14 days of culturing. The metabolic levels of cells cultured on the disassembled shell (10,000 cells cm^−1^) and core (10,000 cells cm^−1^) portions of the tendon scaffold, raw PCL film (as the control of scaffold shell portion; 10,000 cells cm^−1^) and fiber mesh (as the control of scaffold core portion; 10,000 cells cm^−1^), and tissue culture plate (10,000 cells cm^−1^; Thermo Fisher Scientific, USA) were also investigated. Six distinct replicates were tested for each group at all time points.

The viability of human tenocytes seeded in the as-fabricated tendon scaffold was examined using a confocal laser microscopy (FV1000, Olympus, Japan) (*44*). Briefly, after 14 days of culture, cells (seeded with 10,000 cells per sample) were incubated with fluorescein diacetate (FDA; 5 μg μl^−1^ in PBS, 10 min) and, subsequently, with propidium iodide (PI; 4 μg μl^−1^ in PBS, 5 min) at room temperature. After washing thrice with PBS, the scaffold samples were disassembled into separated shell and core portions for imaging immediately. The live and dead cells were identified as FDA-labeled green and PI-labeled red colors, respectively.

To characterize the organization and morphology of live cells, human tenocytes (10,000 cells cm^−1^; sample size, 1 × 1 cm^2^) were seeded on the disassembled shell and core portions of the tendon scaffold and cultured for predetermined periods (1, 3, 7, and 14 days). Cells were fluorescence-labeled with FDA (5 μg μl^−1^ in PBS, 10 min; Sigma-Aldrich), and after washing thrice with PBS, they were imaged immediately using a confocal microscopy. The images of subconfluent cells were analyzed using the built-in function of ImageJ software (NIH, USA). All cells in contact with other cells and image edges were manually removed from the datasets toward single-cell analysis. Cellular angle was determined as the direction of the major elliptic axis of individual cell, as reported previously (*44*). A preferential cell orientation was determined and set as 0°, and cell angles were then normalized to the preferential cell orientation. The number of cells within each degree from −90° to +89° was calculated and normalized such that the total sum was unity. An isotropic sample would be expected to have an even distribution of cell angles in each degree (the percentage of cell angles = 1 × 180^−1^ × 100%). Cells with angles to fall in ±15° were considered to be aligned. Cell elongation was determined by a CSI (circularity = 4 × π × area × perimeter^−2^), with a value of 1 representing the circle. Cells cultured on the raw PCL film (as the control of shell portion; 10,000 cells cm^−1^) and fiber mesh (as the control of core portion; 10,000 cells cm^−1^) were also studied. Images were taken at three to four random regions of each sample for data analysis. Three distinct replicates were tested for each group at all time points.

To analyze cell cytoskeleton and nucleus, human tenocytes cultured at the predetermined periods were fixed with paraformaldehyde [3.7 weight % (wt %) in PBS, 15 min; Sigma-Aldrich], permeabilized using Triton X-100 (0.1 wt % in PBS, 5 min; Sigma-Aldrich), and blocked with bovine serum albumin (2 wt % in PBS, 30 min; Sigma-Aldrich). Cells were then incubated with tetramethyl rhodamine isothiocyanate–conjugated phalloidin (1:200 dilution in PBS; Sigma-Aldrich) and 4′,6-diamidino-2-phenylindole (DAPI; 1:1000 dilution in PBS; Sigma-Aldrich) for the labeling of F-actin filaments and nucleus DNA, respectively. Confocal images were taken and analyzed using the built-in function of ImageJ software (NIH, USA). Cell nuclei with angles falling in the range of ±15° were considered to be aligned. Nucleus elongation was described using an NSI, with a value of 1 representing the circle. Images were taken at three to four random regions of each sample for data analysis. Three distinct replicates were tested for the different groups at each time point.

The major matrix protein (COL-I) and regulating proteins (TNC and DCN) of human tendon were examined using immunofluorescence. Cells were seeded (10,000 cells cm^−1^) on the disassembled shell and core portions (1 × 1 cm^2^) of the tendon scaffold. The raw PCL film and fiber mesh (1 × 1 cm^2^) were set as the controls of the shell and core portions, respectively. After 7 days of culturing, the cells were treated for fixation, permeabilization, and blocking purposes. Cells were then incubated with the mouse-derived antihuman primary monoclonal antibodies [SC-59772 (COL-I), SC-25328 (TNC), and SC-73896 (DCN), all from Axil Scientific; table S2] as testing groups and related normal mouse IgG isotypes as negative controls (SC-3877, Axil Scientific; table S2) at room temperature for 60 min. The cells were then washed thrice with PBS and incubated with the fluorescence-labeled second goat anti-mouse IgG (H + L) antibody (A-11005, Thermo Fisher Scientific; table S2) at room temperature for 60 min. After washing thrice with PBS, the cells were further incubated with DAPI for nucleus visualization. The cells were finally washed thrice with PBS and imaged using a confocal microscopy with the identical parameters for all the groups of each testing marker.

The major protein of human tendon matrix was determined quantitatively on the basis of the measurement of COL-I C-terminal propeptide (CICP) using the MicroVue EIA Kit (mouse-derived antihuman antibodies: MK101, Quidel). Briefly, tenocytes (10,000 cells cm^−1^) were cultured on the disassembled shell and core portions (1 × 1 cm^2^) of the tendon scaffolds for 3, 7, and 14 days. The raw PCL film and fiber mesh were set as the control. For the culturing period of days 0 to 7, the cell medium was changed at days 3 and 7 and kept at a total volume of 1 ml. For the culturing period of days 7 to 14, the addition of 1 ml of new medium was performed at day 10 with a final volume of 2 ml. To measure CICP expression, the collected cultured medium was diluted with an assay buffer (1:12, v/v) and added (100 μl per well) to 96-well stripwells (murine monoclonal anti-CICP antibody coating) for 2 hours of incubation at room temperature. After washing thrice with a wash buffer, the stripwells were added with polyclonal rabbit anti-CICP antibody (100 μl per well) and incubated at room temperature for 45 min. The stripwells, after washing thrice, were then incubated with goat anti-rabbit IgG antibody conjugated to alkaline phosphatase (100 μl per well; room temperature) for 45 min. Last, after washing thrice, the stripwells were incubated with substrate solution containing p-nitrophenyl phosphate (100 μl well^−1^; 30 min), followed by the addition of stop solution (50 μl per well). A series of known concentrations (CICP: 0 to 74.3 ng ml^−1^) were used for the preparation of a standard curve. Immunofluorescence detection was performed at 405 nm using the microplate reader, and the concentration of CICP in culture medium was obtained according to the predetermined standard curve. Cumulative CICP production (CCP) was calculated using an equation as follows

$$CCP_{i}(ng)=C_{3}\times V_{3}+\;+C_{i}\times V_{i}$$ (1)

where *C*_*i*_ and *V*_*i*_ were the measured CICP concentration and the volume of culture medium after *i* days of culturing (*i* = 3, 7, and 14). The production of COL-I on testing samples was expressed by dividing CCP by sample area, while the level of cells to produce COL-I was expressed by dividing CCP by total cell number. Four distinct replicates were tested for each sample group at all time points.

### Ethics statements and in vivo model

Live animals used in this study were in compliance with animal welfare ethical regulations and were approved by the Institutional Animal Care and Use Committee before experimentation. Five 12-month-old male micropigs (20 to 25 kg, more than 1 year old) were obtained from Prestige BioResearch Pte Ltd (Singapore). All animals were fasted for 12 hours before surgery and then sedated with an intramuscular injection of atropine (0.05 mg kg^−1^), ketamine (10 mg kg^−1^), and xylazine (2.5 mg kg^−1^). Surgical procedures were performed under anesthesia using isoflurane inhalation. Briefly, a longitudinal incision was made at the median knee joint from patella to the proximal aspect of tibia in each of the two hind limbs of micropig. The removal of unnecessary connective tissues was performed, and a transected tissue region (2 cm in length) in the midtendon was snipped off to make a full rupture. Two pieces of the tendon scaffolds (gamma sterilization) were implanted side by side to reattach the tendon ends using a modified Kessler technology, as reported previously (*45*). Postoperative medication and monitoring were performed by facility vet daily, and casting bandage was removed 4 weeks after operation. Micropigs were euthanized 1 month (group I, two micropigs) and 3 months (group II, three micropigs) after operation, and implanted samples were collected for histological examination.

### Histology and immunohistochemistry

The harvested samples were fixed immediately in 10% neutral-buffered formalin (24 hours), dehydrated using an alcohol gradient, cleared, and embedded in paraffin blocks, as reported previously (*46*). Histological sections (5 μm in thickness) were prepared using a microtome. H&E staining and Masson’s trichrome staining were performed according to standard procedures to examine the general appearance of soft tissues. The protein expression in the newly formed tissues was examined using immunohistochemistry. The sections were deparaffinized with the Bond Dewax Solution, rehydrated with ethanol gradient, digested with proteinase K to retrieve the antigen, and blocked with goat serum in sequence, according to the standard technical procedures of Institute of Molecular and Cell Biology Advanced Molecular Pathology Laboratory (Singapore). The sections were then incubated with the primary antibodies of monoclonal mouse antihuman COL-I (1:100 dilution in PBS; 15 min) and related IgG1 isotype (1:100 dilution in PBS; negative control, 15 min) and, subsequently, with the secondary polymers (5 min). The slides were finally counterstained with hematoxylin (7 min). The native tendon tissue samples of micropigs were also examined as positive controls for comparison. Samples were imaged using an optical microscope with the identical parameters for each staining.

### Statistical analysis

Data analysis was performed on Prism 6 software and expressed as the means ± SD unless otherwise specified. Statistical comparisons between two groups were based on Student’s paired *t* test with two-tailed distribution. A value of *P* < 0.05 was considered to be statistically significant.

## Supplementary Material

http://advances.sciencemag.org/cgi/content/full/4/10/eaat4537/DC1

## SUPPLEMENTARY MATERIALS

Supplementary material for this article is available at http://advances.sciencemag.org/cgi/content/full/4/10/eaat4537/DC1 Fig. S1. Polymer axial drawing for through-hole expansion and microridge/groove propagation. Fig. S2. Polymer axial drawing for fiber reorientation and deformation. Fig. S3. Cytoskeletal organization and nucleus morphology of human tenocytes. Fig. S4. Human tenocytes to express minor tendon matrix proteins. Fig. S5. Cross section of tendon neotissue construct. Table S1. Mechanical properties of thermally stretched PCL film tube. Table S2. Compiled list of monoclonal antibodies targeted for human tendon matrix markers. Movie S1. Micropigs at 1 month after operation.
