# Supplementary material for: Functional regeneration of tendons using scaffolds with physical anisotropy engineered via microarchitectural manipulation
Source: Sci Adv. 2018 Oct 19;4(10):eaat4537. doi: 10.1126/sciadv.aat4537 (PMC6195336; doi:10.1126/sciadv.aat4537)
Supplement: http://advances.sciencemag.org/cgi/content/full/4/10/eaat4537/DC1 [file supp_4_10_eaat4537__index.html]

Science Advances | Science Advances

## Supplementary Materials

**The PDF file includes:**

- Fig. S1. Polymer axial drawing for through-hole expansion and microridge/groove propagation.
- Fig. S2. Polymer axial drawing for fiber reorientation and deformation.
- Fig. S3. Cytoskeletal organization and nucleus morphology of human tenocytes.
- Fig. S4. Human tenocytes to express minor tendon matrix proteins.
- Fig. S5. Cross section of tendon neotissue construct.
- Table S1. Mechanical properties of thermally stretched PCL film tube.
- Table S2. Compiled list of monoclonal antibodies targeted for human tendon matrix markers.

Download PDF

**Other Supplementary Material for this manuscript includes the following:**

- Movie S1 (.mp4 format). Micropigs at 1 month after operation.

**Files in this Data Supplement:**

- Adobe PDF - aat4537\_SM.pdf
