# Supplementary material for: Functional regeneration of tendons using scaffolds with physical anisotropy engineered via microarchitectural manipulation
Source: Sci Adv. 2018 Oct 19;4(10):eaat4537. doi: 10.1126/sciadv.aat4537 (PMC6195336; doi:10.1126/sciadv.aat4537)
Supplement: http://advances.sciencemag.org/cgi/content/full/4/10/eaat4537/DC1 [file aat4537_SM.pdf]

Science Advances
AAAS

advances.sciencemag.org/cgi/content/full/4/10/eaat4537/DC1

Supplementary Materials for

Functional regeneration of tendons using scaffolds with physical anisotropy engineered via microarchitectural manipulation

Z. Wang*, W. J. Lee, B. T. H. Koh, M. Hong, W. Wang, P. N. Lim, J. Feng, L. S. Park, M. Kim, E. S. Thian*

*Corresponding author. Email: wangzy@hnu.edu.cn (Z.W.); mpetes@nus.edu.sg (E.S.T.)

Published 19 October 2018, *Sci. Adv.* **4**, eaat4537 (2018)
DOI: 10.1126/sciadv.aat4537

The PDF file includes:

Fig. S1. Polymer axial drawing for through-hole expansion and microridge/groove propagation.
Fig. S2. Polymer axial drawing for fiber reorientation and deformation.
Fig. S3. Cytoskeletal organization and nucleus morphology of human tenocytes.
Fig. S4. Human tenocytes to express minor tendon matrix proteins.
Fig. S5. Cross section of tendon neotissue construct.
Table S1. Mechanical properties of thermally stretched PCL film tube.
Table S2. Compiled list of monoclonal antibodies targeted for human tendon matrix markers.

Other Supplementary Material for this manuscript includes the following:

(available at advances.sciencemag.org/cgi/content/full/4/10/eaat4537/DC1)

Movie S1 (.mp4 format). Micropigs at 1 month after operation.

SUPPLEMENTARY MATERIALS

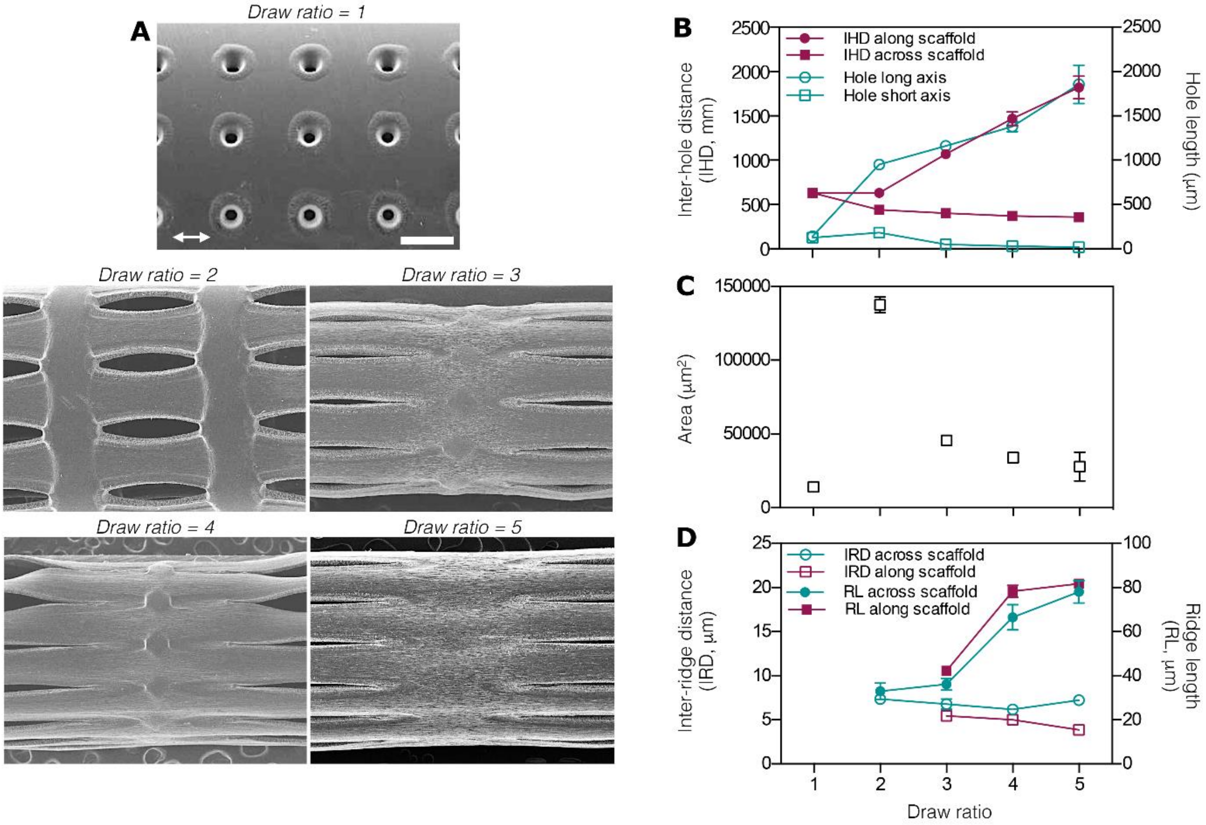

Fig. S1. Polymer axial drawing for through-hole expansion and microridge/groove propagation. (**A**) Representative SEM images of PCL tubes showing a dynamical structure formation during polymer deformation. Scale bar, 500 µm. (**B**) Inter-hole distance (IHD, *N* = 20) and hole size (*N* = 8~20) showing PCL tube elongation as a result of the increase in IHD and hole length along stretching direction, and PCL tube necking associated with the reduction in IHD and hole width across stretching direction. (**C**) Quantitative measurement of through-hole area as a function of draw ratio (*N* = 8~20). (**D**) Inter-ridge distance (IRD, *N* = 60) and ridge length (RL, *N* = 40) coupling with shoulder propagation during polymer axial-drawing. Error bars represent s.d..

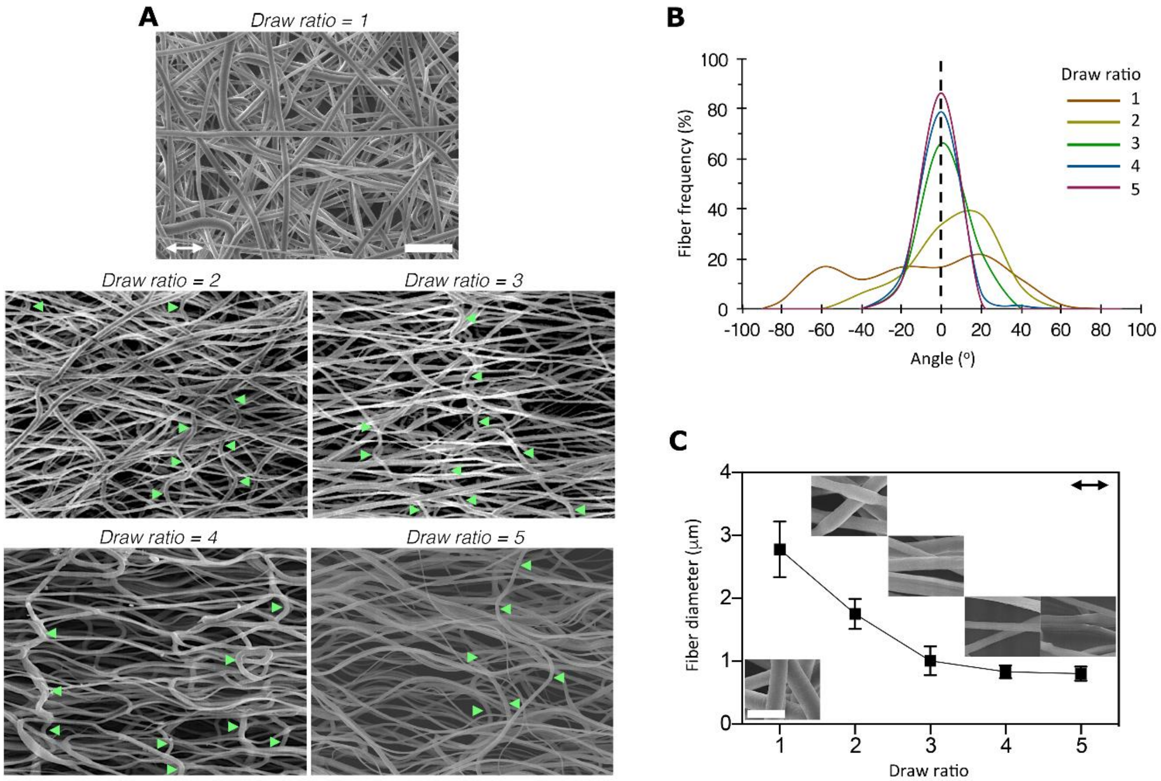

Fig. S2. Polymer axial drawing for fiber reorientation and deformation. (**A**) Representative SEM images of PCL fibre mesh showing a fibre morphological evolution during polymer drawing. Green arrow, loose fibres; and double-headed arrow, stretching direction. Scale bar, 200 μm. (**B**) Representative fibre angular frequency curves showing progressive reorientation of fibres towards stretching direction (dashed line). (**C**) Quantitative measurement of fibre diameter as a function of draw ratio (*N* = 80). Inset, representative SEM images of fibres at draw ratios from 1 to 5; and double-headed arrow, stretching direction. Scale bar, 5 μm. Error bars represent s.d..

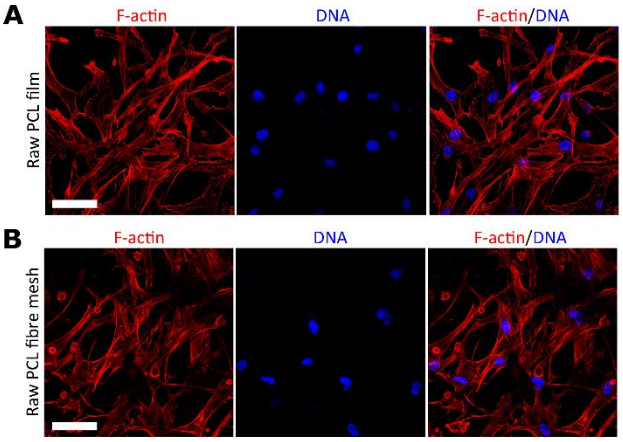

Fig. S3. Cytoskeletal organization and nucleus morphology of human tenocytes. Representative confocal fluorescence images of human tenocytes after 14 days of culturing with raw PCL film (**A**, control of shell portion) and fibre mesh (**B**, control of core portion). Red color, F-actin; and blue color, DNA. Scale bar, 50 μm.

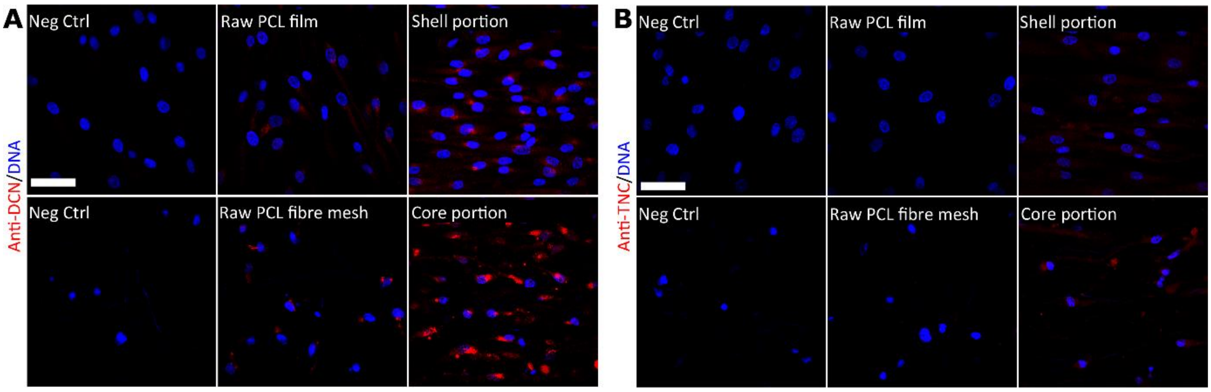

Fig. S4. Human tenocytes to express minor tendon matrix proteins. Representative confocal fluorescence images showing elevated expression of DCN (**A**) and TNC (**B**) for cells after 7 days of culturing with shell (vs. control of raw PCL film) and core (vs. control of raw PCL fibre mesh) portions. DCN, decorin; TNC, tenascin-c; and Neg Ctrl, negative control. Red color, tenogenic marker; and blue color, DNA. Scale bar, 50 µm.

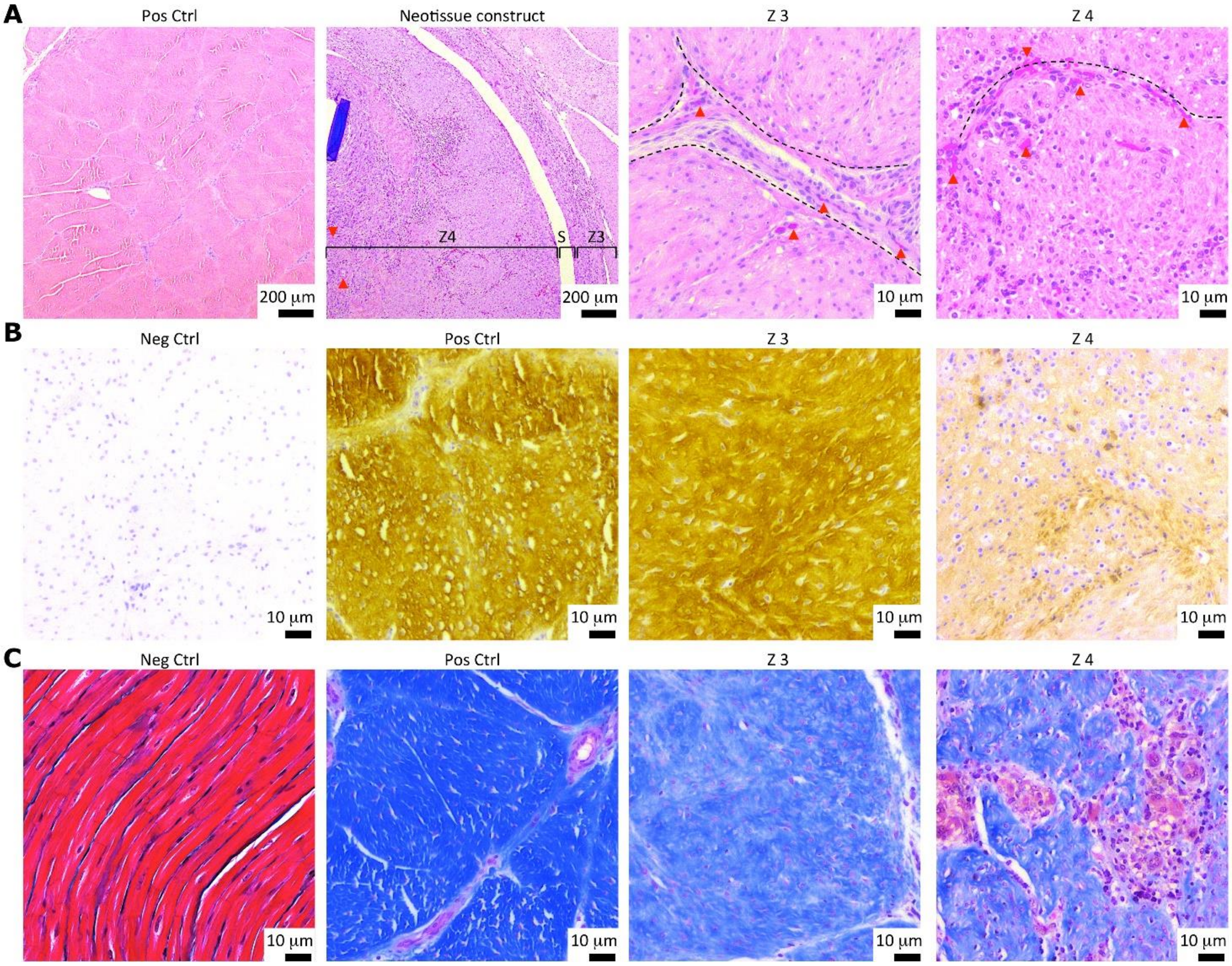

Fig. S5. Cross section of tendon neotissue construct. (**A**) Haemotoxylin and eosin (H&E) images showing full neotissue formation of the scaffold 3 months post operation. s, scaffold edge; z3 and z4, zone of shell and core portions of implanted scaffold; and Pos Ctrl, positive control of native tendon tissue. Red arrow, blood cells and vessels; and dashed line, compartmentalization. (**B**) Immunohistological images showing positive deposition of COL-I matrix in the scaffold. COL-I, collagen type I; Neg Ctrl, negative control of isotype IgG1; and Pos Ctrl, positive control of native tendon tissue. Yellow colour, COL-I; and blue colour, cell nuclei. (**C**) Masson's trichrome images showing fibrous matrix formation in the scaffold, with compartments of hypo- and hyper-cellular regions. Neg Ctrl, negative control of heart tissue; and Pos Ctrl, positive control of native tendon tissue. Blue colour, matrix fibres; red/pink colour, cytoplasm; and dark/purple colour, nuclei.

Table S1. Mechanical properties of thermally stretched PCL film tube. (n=4)

Samples	Young's modulus (MPa)	Yield stress (MPa)	Yield strain (%)	Ultimate stress (MPa)	Break strain (%)
PCL film tube	438.9 ± 37.9	46.4 ± 4.4	11.8 ± 2.0	114.8 ± 8.6	182.7 ± 9.0

*: Fabricated from raw film tube using polymer axial-drawing at a draw ratio of 5 and temperature of 54 °C.

Table S2. Compiled list of monoclonal antibodies targeted for human tendon matrix markers.

Primary monoclonal antibodies (Mouse anti-human)	IgG isotypes	Dilution in 1 wt.% BSA solution (v/v)
COL-I (COL1A1)	IgG 1	1:100
TNC	IgG 1	1:100
DCN	IgG 1	1:100
Second antibody (Goat anti-mouse IgG (H+L)-Alexa Fluor® 594)	/	1:200
